# Threat vs. Threat: Attention to Fear-Related Animals and Threatening Faces

**DOI:** 10.3389/fpsyg.2018.01154

**Published:** 2018-07-23

**Authors:** Elisa Berdica, Antje B. M. Gerdes, Florian Bublatzky, Andrew J. White, Georg W. Alpers

**Affiliations:** Clinical and Biological Psychology and Psychotherapy, Department of Psychology, School of Social Sciences, University of Mannheim, Mannheim, Germany

**Keywords:** attention, emotion, spiders, emotional facial expressions, free viewing

## Abstract

It is generally thought to be adaptive that fear relevant stimuli in the environment can capture and hold our attention; and in psychopathology attentional allocation is thought to be cue-specific. Such hypervigilance toward threatening cues or difficulty to disengage attention from threat has been demonstrated for a variety of stimuli, for example, toward evolutionary prepared animals or toward socially relevant facial expressions. Usually, specific stimuli have been examined in individuals with particular fears (e.g., animals in animal fearful and faces in socially fearful participants). However, different kinds of stimuli are rarely examined in one study. Thus, it is unknown how different categories of threatening stimuli compete for attention and how specific kinds of fears modulate these attentional processes. In this study, we used a free viewing paradigm: pairs of pictures with threat-related content (spiders or angry faces) or neutral content (butterflies or neutral faces) were presented side by side (i.e., spiders and angry faces, angry and neutral faces, spiders and butterflies, butterflies and neutral faces). Eye-movements were recorded while spider fearful, socially anxious, or non-anxious participants viewed the picture pairs. Results generally replicate the finding that unpleasant pictures more effectively capture attention in the beginning of a trial compared to neutral pictures. This effect was more pronounced in spider fearful participants: the higher the fear the quicker they were in looking at spiders. This was not the case for high socially anxious participants and pictures of angry faces. Interestingly, when presented next to each other, there was no preference in initial orientation for either spiders or angry faces. However, neutral faces were looked at more quickly than butterflies. Regarding sustained attention, we found no general preference for unpleasant pictures compared to neutral pictures.

## Introduction

Humans are exposed to a plethora of concurrent visual stimuli and attention is preferentially directed to the most menacing of them. This is thought to be adaptive because quick detection of danger promotes survival by initiating necessary behavioral responses (Öhman et al., 2001). The selective allocation of attention toward a particular type of stimulus is referred to as attentional bias. Among more fearful individuals, this bias tends to be particularly strong (Öhman and Mineka, 2001; Mineka and Öhman, 2002). Furthermore, attentional biases are generally thought to be specific for particular fears: For example, specific phobias are related to pictures of the feared animals, social anxiety is related to negative (or neutral) emotional facial expressions (Cisler and Koster, 2010).

Among all the visual stimuli that are encountered, faces are especially relevant social cues for us (Alpers and Gerdes, 2007; Bublatzky and Alpers, 2017). Accordingly, humans are equipped with distributed neural systems that specialize in face processing (Haxby et al., 2000). Substantial evidence shows that faces are processed more efficiently than other neutral stimuli (Farah et al., 1998; Öhman et al., 2001) by facilitating perceptual and attentional processing (Alpers and Gerdes, 2007; Alpers et al., 2011; Bublatzky et al., 2014). Similarly specialized neural systems are likely involved in the preferential processing of other kinds of evolutionary significant stimuli. It has thus been suggested that humans are equipped with a preparedness for specific animals (i.e., spiders, snakes) that may be particularly relevant for our survival (Seligman, 1971). Indeed, compelling evidence indicates that fear-related animals are preferentially processed (Macleod and Mathews, 1988), which manifests itself in eye-tracking research as longer fixation durations or as greater skin conductance responses compared to neutral stimuli (Gerdes et al., 2008, 2009a; Wiemer et al., 2013). Still, the stimuli used in such studies are typically very diverse, e.g., potentially threatening animals like snakes, spiders, insects (Lipp, 2006; Rinck and Becker, 2006; Gerdes et al., 2008, 2009b; Weymar et al., 2013; Berdica et al., 2014, 2017).

Although different kinds of threatening stimuli (fear-related animals and social stimuli) evoke comparable reactions compared to neutral stimuli, this does not mean that all threatening stimuli are the same. For example, in an earlier study we demonstrated that pictures of emotional scenes and pictures of faces can result in very different psychophysiological response patterns (Alpers et al., 2011). Moreover, data from event-related potentials in another study (Kolassa et al., 2008) show enhanced attention to emotional face stimuli in socially anxious individuals regardless of facial expression; this was not specific to social phobia but was also observed in those participants who were afraid of spiders. No abnormalities were found in the processing of angry faces in particular (Kolassa et al., 2008). The authors also found that spider phobic individuals were generally more hypervigilant toward any kind of stimuli, suggesting a generalization of anxious behavior in spider phobic individuals. This finding is supported by previous research (Weymar et al., 2013) which showed an enhanced hypervigilance for all of the stimuli that they presented (both spiders and butterflies) in spider fearful participants.

Similar response patterns to different evolutionary fear-relevant stimuli are noticeable in infants younger than 9 months (Erlich et al., 2013). In this study, human voices and animal sounds (hissing snakes) were considered as part of the evolutionary fear-relevant category and they elicited similar reactions. At the same time, fear responses to threatening animal stimuli or to emotional facial expressions can differ in many ways, in terms of physiological or behavioral responses, fear learning, or fear conditioning. For example, in a recent review it was shown that fear conditioning to social stimuli (angry faces) was shown to be less robust as fear conditioning to snake and spider stimuli. The authors proposed that distinct mechanisms may underlie fear learning for social and animal stimuli (Mallan et al., 2013). In terms of brain activation, in a recent EEG study Langeslag and van Strien (2018) found that snakes capture attention faster than angry faces, manifested in more pronounced EPN for snakes compared to angry faces. In this study, pictures were presented separately in rapid serial visual presentation therefore no conclusions about initial orienting of attention when both pictures are presented side by side can be reached.

Hence, there is considerable evidence that both, pictures of threatening animals and of emotional facial expressions trigger fear responses, avoidant behavior, they differ in fear learning and in fear conditioning. Öhman (2009) postulate a predatory defense system related to evolutionary prepared animals like snakes, and a social submissiveness system corresponding to social fears. They support the hypothesis that these sorts of stimuli would elicit fear after minimal processing times (Öhman, 2009). There is, however, a lack of studies in which attentional processing of threatening animals and faces are directly compared. Faces have, nonetheless been compared with pictures of other emotional scenes before (Alpers et al., 2011; Eisenbarth et al., 2011), but not specifically with threatening animals like snakes or spiders.

Furthermore, no studies so far have addressed the specificity of attentional biases – is there a uniform bias to emotionally relevant pictures or is there a category specific bias? Are specific categories more dominant than others? Until now, the question how these types of stimuli compete for visual attention and whether specific fear (e.g., spider vs. face) can differentially modulate the underlying mechanisms of attentional biases remains still unanswered. In order to answer these questions, the main purpose of the present study was to examine whether angry faces and spiders elicit similar attentional biases (initial vigilance; difficulties to disengage).

Another important aspect we deal with in the present research is the time course of attention allocation to fear-related stimuli. This has also been tested in one *or* the other stimulus type (Animals: Lipp, 2006; Rinck and Becker, 2006; Gerdes et al., 2008, Faces: Mogg et al., 2004) but never for both types placed next to each other in one single experiment.

There is considerable evidence to support the notion that attentional biases are comprised of an initial hypervigilance for threat (Mogg and Bradley, 1998; Öhman, 2009). How aversive stimuli are subsequently processed — whether these stimuli are avoided or attended more — is less clear. Some researchers in the field suggest that an initial orienting toward threat is followed by a difficulty in disengaging from threat (Fox et al., 2001, 2002; Gerdes et al., 2008, 2009a). In contrast, others suggest that initial hypervigilance is followed by avoidant behavior (Mathews and Mackintosh, 1998; Mogg and Bradley, 1998; Wieser et al., 2009) making it challenging to reach conclusions on sustained attention on threatening stimuli.

Face stimuli are frequently used to investigate the aforementioned attentional processes. For example, in an eye-tracking study, Holas et al. (2014) found an initial vigilance for angry and happy faces, but at the same time inconsistent results for the sustained attention. Likewise, in another study, initial hypervigilance for angry faces was found but no subsequent bias in later processing (Gamble and Rapee, 2010). Furthermore, a recent study found no initial orienting bias in socially anxious individuals toward threat but a difficulty to disengage attention from threatening stimuli once their initial attention is oriented toward those stimuli (Liang et al., 2017).

Given the contradictory models for the sustained attention mentioned above, as well as the limited range of examined stimuli used in previous experiments, in the present study we directly compared faces and animals in a typical free viewing paradigm to identify attentional biases (in anxiety). Pairs of emotional (spiders, angry faces) and neutral (butterflies, neutral faces) visual stimuli were presented in one of several combinations: pictures of spiders and butterflies, angry and neutral faces, spiders and angry faces, butterflies, and neutral faces.

Taken together, the main purpose of our study was to examine whether spiders and angry faces elicit similar attentional biases (initial vigilance; difficulties to disengage) when directly compete for attentional resources. In order to achieve this, we use eye-tracking as an advantageous measure to investigate these attentional biases (Armstrong and Olatunji, 2012). We further aimed at replicating the preference toward threat hypothesis: spiders and angry faces should generally be detected faster than butterflies and neutral faces. Based on the findings reported above, spiders, and angry faces were not expected to differ in initial orienting of attention when pitted against each other but faces were expected to sustain attention longer compared to animal stimuli for all participants independent from specific fear. This pattern was expected to be enhanced for socially anxious individuals.

## Materials and Methods

### Participants

Seventy participants (11 men) were recruited via newspaper advertisements from the general population and from the student population of the University of Mannheim. Their age ranged from 18 to 51 years (*M* = 27.21, *SD* = 8.98). All participants reported normal or corrected-to-normal vision, were naive to the purpose of the experiment and gave written informed consent prior to participation. The unselected sample scored within the normal range of spider phobia and social anxiety with respect to normative samples (Rinck et al., 2002; Sosic et al., 2008) on these self-report measures. After the experiment, detailed information on spider/social fear was provided if requested and students received course credits.

### Stimulus Material and Design

**FIGURE 1:**
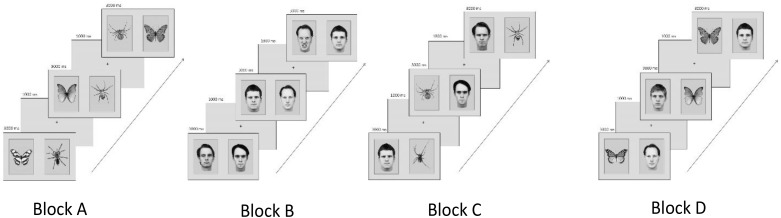
Examples of experimental category combinations, trial sequences, and block order counterbalanced across participants. (Block A) Spiders and butterflies, (Block B) angry and neutral faces, (Block C) spiders and angry faces, and (Block D) butterflies and neutral faces. The codes for the KDEF faces used in the figure are: am10nes, am10ans, am21ans, am21nes, am23nes, am24ans, bm10nes, bm23nes, bm31ans, and bm31nes.

Thirty-two pictures of male angry and neutral faces from the Karolinska Directed Emotional Faces set (Lundqvist et al., 1998) were used as face stimuli. In addition, spiders and butterflies (black and white; adjusted for contrast and brightness) were selected from previous studies (e.g., Gerdes et al., 2009a). Pictures were presented in pairs of two displaying each (1) a spider and a butterfly, (2) an angry and a neutral face, (3) a spider and an angry face, and (4) a butterfly and a neutral face (**Figure 1**).

### Procedure

After arrival at the laboratory, participants completed a set of questionnaires. To check for fear of spiders we used the FAS (German version of the Fear of Spiders Questionnaire; FSQ: Szymanski and William, 1995; German version: Rinck et al., 2002) and for social anxiety the SPIN (SPIN: Connor et al., 2000; German version: Sosic et al., 2008). In addition, the State-Trait Anxiety Inventory (STAI: Spielberger et al., 1983; German version: Laux et al., 1981) was used to assess the level of state anxiety before and after the experiment. The computer task started with 20 practice trials for the participants to get familiar with the experiment followed by the four experimental blocks (counterbalanced order), and picture rating regarding valence and arousal (SAM: Bradley and Lang, 1994). The four picture-category combinations were presented in separate blocks of 64 trials (block order was counterbalanced), which resulted in a total 256 trials. Each trial consisted of a 1 s fixation cross serving as an Inter Trial Interval (ITI) followed by a 3 s picture presentation. Pictures (562 × 762 pixels) were presented on a 22 inch monitor (1024 × 768 pixels) about 50 cm away from the participant.

Participants were instructed to simply look at the pictures that appear on the screen. The procedures of the experiment were approved by the ethics committee of the University of Mannheim.

### Data Recording and Reduction

Eye movements were recorded with an SMI RED250 eye-tracking device (SMI, Teltow, Germany), which has a sampling rate of 250 Hz and tracking resolution of 0.03°. A frame around each picture was the area of interest (AOI) for the analysis in BeGaze (SMI, Teltow, Germany).

Trials without saccades, or without proper fixation on the fixation cross, were deleted from further analyses. We then calculated the entry viewing time for each picture. Entry time was defined as the average duration after picture onset when the first fixation on the picture was recorded. Second, we calculated the number of fixations (fixation count) as an index of the total time that participants spent looking at a specific picture (number of all fixations for all subjects divided by number of subjects).

### Data Analyses

Self-report data were analyzed with separate 2 × 2 ANOVAs for the mean valence and arousal ratings with Emotion (fear-related vs. neutral) and Type of Stimuli (animals vs. faces) as within subject’s factor. In a second step questionnaire scores of fear of spiders and social anxiety were included separately as covariates in ANCOVA analyses. Afterward, we conducted *post hoc* comparisons of the correlations. One participant did not complete the rating therefore we report only the rating data of 69 participants here.

More specifically we analyzed entry time with a 2 × 2 analysis of variance (ANOVA) for the four different block types (spiders and butterflies, angry and neutral faces, spiders and angry faces, and butterflies and neutral faces), with the factors Position of each picture (left or right) and Emotion (fear-related or neutral) for the first two blocks, and Position of each picture (left or right) and Type of Stimuli (animal stimuli or face stimuli) for the last two blocks. Furthermore, separate analyses of covariance (ANCOVA) including the questionnaire scores of fear of spiders and social anxiety as covariate were conducted to investigate the influence of individual differences (spider fear and social anxiety). We used an ANCOVA so that we had no loss in power (Royston et al., 2006). Moreover, as a *post hoc* analysis we compared the correlation coefficients in order to assess the significance of the difference between the correlation coefficients using the Fisher r-to-z transformation (Lenhard and Lenhard, 2014).

We then analyzed the number of fixations index based on a ratio analysis. As fear-related and neutral pictures appeared in pairs, raw data of fixation counts were not independent. Therefore, we computed preference ratios as suggested by Alpers (2008). These ratios were computed as follows: Preference ratio (PR) = number of first fixations on the fear-related picture minus number of first fixations on the neutral picture divided by the sum of the two. Values from 0.5 to 1 indicate a preference for the fear-related over neutral pictures; values ranging from 0 to 0.5 indicate a relative preference for neutral pictures. Values not significantly different from zero described the absence of differences between fear-related relative to neutral pictures. Preference ratios were used as dependent variables in an ANOVA with the within-subject factor Position of the unpleasant picture (left vs. right). Furthermore, we conducted separate ANCOVAs including the questionnaire scores of fear of spiders and social anxiety as covariate. Afterward, we tested whether the grand mean, that is the average preference ratio across all factor levels, is significantly different from 0.5.

Regarding the self-report ratings, we conducted separate ANOVAs for the mean valence and arousal ratings with a 2 × 2 design with Emotion (fear-related vs. neutral) and Type of Stimuli (animals vs. faces) as within subject’s factor.

In order to test the effects of individual’s particular extend of a specific fear/anxiety we then included questionnaire scores of fear of spiders and social anxiety were also included as covariates in an ANCOVA analysis separately^1^. Afterward, we conducted *post hoc* comparisons of the correlation coefficients. One participant did not complete the rating therefore we report only the rating data of 69 participants.

## Results

### Stimulus Ratings

#### Valence Ratings

The ANOVA for the mean valence rating showed a significant main effect of Emotion, *F*(1,68) = 414.6, *p* < 0.001, η^2^ = 0.86, and a significant main effect of Stimulus Type *F*(1,68) = 13.5, *p* < 0.001, η^2^ = 0.16. Overall, spider pictures were rated as more negative (*M* = 7.17, *SD* = 1.20) than butterflies (*M* = 3.10, *SD* = 1.28), and angry faces as more negative (*M* = 6.45, *SD* = 1.22) than neutral faces (*M* = 4.99, *SD* = 0.88). This was supported from follow-up *t*-tests: for spiders vs. butterflies valence rating *t*(68) = 49.6, *p* < 0.001; for angry faces vs. neutral faces *t*(68) = 43.73, *p* < 0.001.

In the ANCOVA with fear of spiders as a covariate the main effect of Emotion covaried with fear of spiders, *F*(1,67) = 169.8, *p* < 0.001, η^2^ = 0.71 and so did the Type of Stimulus *F*(1,67) = 12.93, *p* < 0.001, η^2^ = 0.16. As a *post hoc* analysis of valence rating scores and spider pictures (*r* = 0.70) vs. butterflies pictures (*r* = 0.04) resulted in (Z = -4.80, *p* < 0.001) which indicates that the higher the fear of spiders, the more negative all fear-related stimuli were rated (spiders and angry faces). Furthermore, social anxiety did not reveal significant covariation with Emotion *F*(1,67) = 0.05, *p* = 0.82, η^2^ = 0.01 or Stimulus Type *F*(1,67) = 0.37, *p* = 0.54, η^2^ = 0.06.

#### Arousal Ratings

The ANOVA for the mean arousal rating showed a significant main effect of Emotion, *F*(1,68) = 126.2, *p* < 0.001, η^2^ = 0.65 and a significant main effect of Type of Stimulus *F*(1,68) = 9.38, *p* = 0.003, η^2^ = 0.12. Spider pictures were rated as more arousing (*M* = 5.75, *SD* = 2.11) than butterflies (*M* = 2.93, *SD* = 1.58) [*post hoc t*-tests for spiders and butterflies arousal rating: *t*(68) = 22.66, *p* < 0.001] and angry faces as more arousing (*M* = 4.43, *SD* = 1.65) than neutral faces (*M* = 3.05, *SD* = 1.51); [*post hoc t*-tests for angry faces and neutral faces: *t*(68) = 22.28, *p* < 0.001].

Furthermore, in the ANCOVA with fear of spiders as a covariate, there was an interaction of Emotion with fear of spiders *F*(1,67) = 27.84, *p* < 0.001, η^2^ = 0.29 and an interaction of Type of Stimulus and fear of spiders *F*(1,67) = 20.34, *p* < 0.001, η^2^ = 0.23. *Post hoc* analysis: Spiders (*r* = 0.74) vs. butterflies (*r* = -0.02 with comparison of the correlations: (Z = -5.63, *p* < 0.001), suggesting that the higher the fear of spiders, the more arousing participants perceived fear-related pictures (spiders and angry faces).

When we compared spiders with angry faces, spiders were rated as more negative *t*(68) = 3.64, *p* = 0.001 and as more arousing *t*(68) = 4.46, *p* < 0.001 than angry faces. Among the neutral categories, butterflies were rated as more positive than neutral faces *t*(68) = 9.69, *p* < 0.001 but no significant difference was detected for arousal ratings, *t*(68) = 0.691, *p* = 0.49.

Moreover, no interaction of Social Anxiety and Emotion was found *F*(1,67) = 6.26, *p* = 0.11, η^2^ = 0.04, but there was a significant interaction of social anxiety and Type of Stimulus *F*(1,67) = 6.19, *p* = 0.015, η^2^ = 0.08: the higher the social anxiety, the more arousing angry faces were rated; but this effect was not significant when we compared the correlations: angry faces (*r* = 0.36) vs. neutral faces (*r* = 0.29): (Z = -0.42, *p* = 0.34). Interestingly social anxiety influenced the rating of butterflies and neutral faces: butterflies (*r* = -0.11) vs. neutral faces (*r* = 0.29): (Z = -2.37, *p* = 0.009) which suggests that neutral faces were rated as more arousing compared to butterflies.

### Eye-Tracking Data

#### Entry Time on Each Picture

##### Spiders vs. butterflies

**FIGURE 2:**
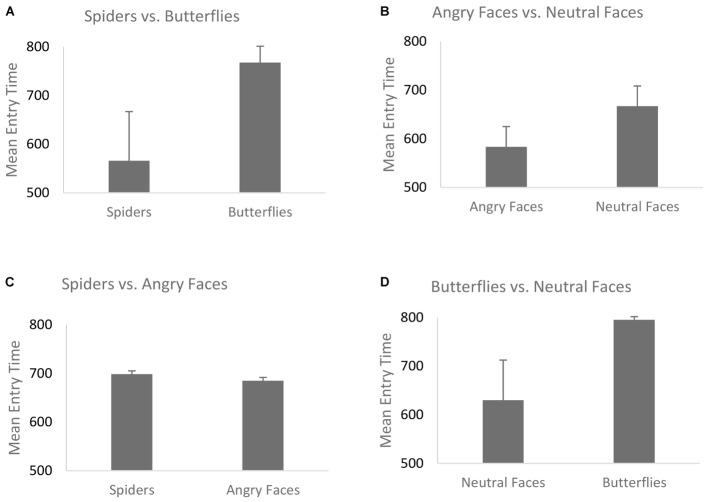
Mean entry time for the four blocks. **(A)** Spiders vs. butterflies; **(B)** angry faces vs. neutral faces; **(C)** spiders vs. angry faces; **(D)** butterflies vs. neutral faces. Error bars represent the standard error of the mean.

**FIGURE 3:**
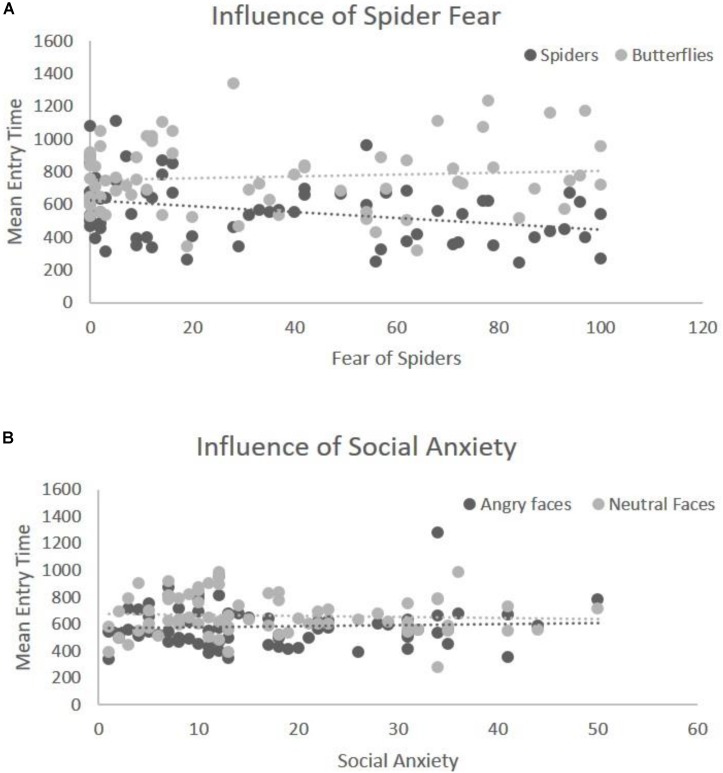
Illustration of covariation between Emotion and fear level. **(A)** Significant interaction of entry time with spider fear for the first block (spiders vs. butterflies). The *y*-axis represents the mean entry time across trials and the *x*-axis represents the questionnaire scores for fear of spiders (range 0–126). Dots represent the type of stimulus and the lines represent the regression lines **(B)** interaction of entry time with social anxiety for the second block (angry faces vs. neutral faces). The *y*-axis represents the mean entry time across trials and the *x*-axis represents the questionnaire scores for social anxiety (range 0–60). Dots represent the type of stimulus and the lines represent the regression lines.

The mean entry time varied as a function of Emotion, *F*(1,68) = 40.29, *p* < 0.001, η^2^ = 0.42, [in Blocks 1 and 2 we only use the factor Emotion, and the Type of Stimulus is held constant – (see the “Data Analyses” section)] indicating that fear-related spider pictures were looked at more quickly than neutral pictures of butterflies: *F*(1,68) = 40.29, *p* < 0.001, η^2^ = 0.42 (**Figure 2B**). Moreover, this effect of Emotion covaried with self-reported fear of spiders, *F*(1,68) = 6.75, *p* = 0.01, η^2^ = 0.05. To follow-up on this interaction we compared the correlations for entry time and fear of spiders: spider pictures (*r* = -0.31) and butterfly pictures (*r* = 0.09). These correlations differed significantly, Z = -2.42, *p* = 0.008, indicating that the higher the fear of spiders, the more pronounced this effect was (**Figure 3B**), i.e., participants with higher fear of spiders look at spider pictures more quickly.

Furthermore, a main effect of Position emerged, *F*(1,69) = 27.61, *p* < 0.001, η^2^ = 0.26, showing more first fixations on pictures appearing on the left compared to the right visual hemifield. However, there was no interaction of Emotion and Position, *F*(1,69) = 0.08, *p* = 0.77, η^2^ = 0.02.

##### Angry vs. neutral faces

In the overall ANOVA with mean entry time and Emotion [again, for Block 2 the Type of Stimulus factor was kept constant (see the “Data Analyses” section)] there was a main effect of Emotion *F*(1,69) = 16.30, *p* < 0.001, η^2^ = 0.22. That is, fear-related stimuli (angry faces) were looked at more quickly than neutral stimuli (neutral faces), (**Figure 2A**). The ANCOVA showed no interaction of this main effect with social anxiety questionnaire scores, *F*(1,68) = 0.81, *p* = 0.37, η^2^ = 0.011 (**Figure 3A**). *Post hoc* correlations for social anxiety with pictures of angry faces (*r* = -0.02) and pictures of neutral face (*r* = -0.05)] resulted in the comparison: (Z = -0.14, *p* = 0.44).

Furthermore, a main effect of Position emerged, *F*(1,69) = 42.02, *p* = 0.00, η^2^ = 0.28, suggesting more first fixations on pictures appearing on the left side. However, there was no interaction of Emotion and Position, *F*(1,69) = 1.26, *p* = 0.26, η^2^ = 0.015.

##### Spiders vs. angry faces

**FIGURE 4:**
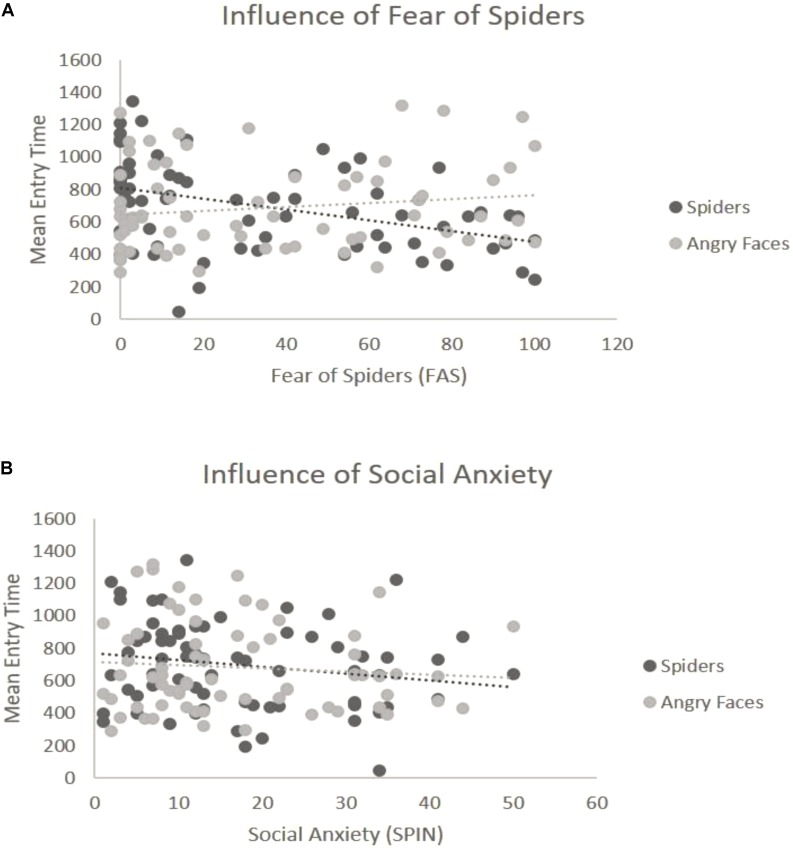
Interaction of emotion with reported levels of fear for the third block (spiders vs. angry faces). **(A)** Significant interaction with spider fear. The *y*-axis represents the mean entry time across trials and the *x*-axis represents the questionnaire scores for fear of spiders (range 0–126). Dots represent the type of stimulus and the lines represent the regression lines. **(B)** Non-significant interaction with social anxiety. The *y*-axis represents the mean entry time across trials and the *x*-axis represents the questionnaire scores for social anxiety (range 0–60). Dots represent the type of stimulus and the lines represent the regression lines.

For the overall ANOVA with mean entry time and Type of Stimulus [in Blocks 3 and 4 the Emotion factor was held constant (see “Data Analysis” section)] was not found to have an effect on the entry time — we found no differences between spiders and angry faces, *F*(1,69) = 0.06, *p* = 0.8, η^2^ = 0.04 (**Figure 2C**). At the ANCOVA the Stimulus Type covaried with spider fear, *F*(1,68) = 9.51, *p* = 0.003, η^2^ = 0.14. *Post hoc* correlation comparison analysis for fear of spiders and spider pictures: (*r* = -0.42) and *post hoc* correlation comparison for social anxiety and angry face pictures (*r* = 0.15): (Z = -3.49, *p* < 0.01). This indicates that the higher the fear of spiders, the quicker participants were to look at spider pictures (**Figure 4A**). No such relationship was found with social anxiety and pictures of angry faces, *F*(1,68) = 0.25, *p* = 0.61, η^2^ = 0.05 (**Figure 4B**), spiders (*r* = -0.19) and angry faces (*r* = -0.09) and comparison (Z = -0.59, *p* = 0.27). In addition, a main effect of Position emerged, *F*(1,69) = 29.27, *p* < 0.001, η^2^ = 0.37, such that there were more first fixations on pictures that appeared on the left side.

##### Butterflies vs. neutral faces

The mean entry time varied as a function of Type of Stimulus [(in Block 4, same like in Block 3, Emotion factor was held constant (see “Data Analysis” section)] suggesting that neutral faces are looked at faster than butterflies, *F*(1,69) = 10.02, *p* = 0.002, η^2^ = 0.15 (**Figure 2D**). No interaction with fear of spiders was found *F*(1,68) = 3.12, *p* = 0.08, η^2^ = 0.02 [*post hoc* analysis fear of spiders for butterfly pictures (*r* = -0.04) and neutral face pictures (*r* = -0.19) resulted at the comparison (Z = 0.9, *p* = 0.18)]. The ANCOVA with social anxiety as a covariate *F*(1,68) = 0.12, *p* = 0.72, η^2^ = 0.08, also did not correlate with social anxiety scores [*post hoc* analysis for social anxiety and butterfly pictures (*r* = -0.28) and neutral face pictures (*r* = -0.05) resulted at the comparison (Z = -1.34, *p* = 0.09)]. Moreover there was also a main effect of Position, *F*(1,69) = 33.05, *p* < 0.001, η^2^ = 0.28, such that entry time was shorter for pictures appearing on the left side.

#### Number of Fixations on Each Picture

##### Spiders vs. butterflies

The overall ANOVA for the preference ratio of number of fixations with the within-subject factor Position did not reveal any main effects: *F*(1,68) = 0.41, *p* = 0.52, η^2^ = 0.02. For the number of fixations no overall preference was found for the spiders (*M* = 135) compared to the butterflies (*M* = 134) – positive grand mean [*M* = 0.49, *SD* = 0.02, *t*(69) = -0.27, *p* = 0.78]. The ANCOVA with fear of spiders as a covariate showed no interaction with fear of spiders, *F*(1,68) = 0.34, *p* = 0.56, η^2^ = 0.02.

##### Angry vs. neutral faces

The overall ANOVA for the preference ratio of number of fixations with the within-subject factor Position did not reveal any main effects: *F*(1,68) = 0.67, *p* = 0.41, η^2^ = 0.004. Number of fixations was not different for fear-related stimuli angry faces (*M* = 155) and neutral face (*M* = 145): positive grand mean [*M* = 0.52, *SD* = 0.01, *t*(69) = 1.9, *p* = 0.61]. Furthermore, there was no covariation with social anxiety scores, *F*(1,68) = 1.98, *p* = 0.16, η^2^ = 0.05.

##### Spiders vs. angry faces

The preference ratio of the number of fixations did not vary as a function of Position: *F*(1,68) = 0.005, *p* = 0.94, η^2^ = 0.04. The number of fixations was not significantly different for fearful animal stimuli (spiders, *M* = 127) and angry faces (*M* = 153): positive grand mean [*M* = 0.45, *SD* = 0.02, *t*(69) = -2, *p* = 0.051]. No interaction with fear of spiders was found *F*(1,68) = 0.17, *p* = 0.67, η^2^ = 0.005, and likewise no interaction with social anxiety scores *F*(1,68) = 0.62, *p* = 0.73, η^2^ = 0.002.

##### Neutral faces vs. butterflies

The overall ANOVA for the preference ratio of number of fixations with the within-subject factor Position did not reveal any main effects: *F*(1,68) = 0.37, *p* = 0.54, η^2^ = 0.03. There was no overall preference for the neutral faces (*M* = 146) compared to the butterflies (*M* = 138): positive grand mean [*M* = 0.51, *SD* = 0.02, *t*(69) = -0.92, *p* = 0.36]. No interaction with fear of spiders was found when used as a covariate, *F*(1,68) = 0.37, *p* = 0.54, η^2^ = 0.04. Likewise no interaction was found in the ANCOVA with social anxiety, *F*(1,68) = 0.37, *p* = 0.54, η^2^ = 0.04.

## Discussion

Attentional biases have been documented for fear-related animals (such as spiders) and emotional facial expressions (such as angry faces) but they have rarely been pitted against each other in one experiment. This is the first eye-tracking study to directly compare how threatening animal stimuli and social stimuli compete for visual attention. We recorded eye-movements during a free viewing task with pictures from different categories (animals and human faces), displaying emotional (spiders, angry faces) or neutral (butterflies, non-emotional faces) information. Our findings support an attentional preference toward threat, thus, this replicates previous findings in a more complex context. Overall, fear-related stimuli captured initial attention more than neutral pictures. As for the comparison between categories (animal vs. faces), there were no obvious differences in the initial allocation of attention or in sustained attention.

Eye-tracking methodology provides a useful tool to investigate attentional biases (see Armstrong and Olatunji, 2012); it does, in addition measure different attentional processes within different time frames: initial hypervigilance in early stages of processing and sustained attention in later stages. For this reason, we focused our analysis on two parameters: Entry time as an indicator of initial hypervigilance and fixation count as an indicator of sustained attention. The first parameter taps into the early component of the hypervigilance-avoidance hypothesis (Mogg and Bradley, 2006). Our results support the notion that unpleasant stimuli capture attention faster than neutral stimuli—threatening pictures were generally looked at faster than neutral pictures. In addition to this, our design also allows for a direct comparison of entry times in a single experimental design. This is in line with brain imaging data that show that negative emotional stimuli are preferentially processed in the brain (Alpers et al., 2009; Gerdes et al., 2010).

In addition, typical left bias was also observed in our study, which may represent the common reading direction. Pictures on the left were detected faster than the ones on the right, independent of stimulus type. A left bias for processing emotional information has been found before (Alpers, 2008) and it would have been expected but it was not supported from the current data.

Regarding sustained attention, fixation count data do not differ for spiders or faces. More fixations were observed for face stimuli compared to spider pictures in Block 3 but this did not reach significance. Similar to other studies (Calvo and Lang, 2004), we also did not find an emotion effect regarding fixation count — fear-related pictures did not hold attention longer than neutral stimuli. Furthermore, a left bias was also absent for the sustained attention parameter. It should be noted that a difficulty to disengage from threat (or sustained attention) may be visible only during visual search tasks but not during free viewing tasks (Armstrong and Olatunji, 2012). Therefore, task relevance should be investigated further in future studies.

Regarding the neutral category, butterflies were rated as more positive than neutral faces which can also be seen in the normative ratings of the few pictures depicting butterflies in the IAPS collection (Lang et al., 1999). One reason for this result might be the fact that butterflies are more unambiguous pictures while neutral faces sometimes appear ambiguous and tend to sometimes be regarded as negative (Melfsen and Florin, 2002; Blasi et al., 2009). Future research should investigate the extent to which attentional processes are similar for different types of neutral stimuli.

One reason for the differences in eye movements in participants with fear of spiders and social anxiety could be that social anxiety is generally considered to be more complex than specific phobias. Attentional biases in general in fear of spiders can be explained in terms of preparedness while social anxiety can be seen as a result of perceived dominance (Öhman, 2009). Interestingly, animal fears are relevant between species, while social phobia within-species. Moreover, while specific phobias are centered on a specified object, social anxiety comprises, among everything else, fear of criticism or disapproval (Hofmann et al., 2009). A large body of research documents attentional biases in individuals with specific animal fear (Gerdes et al., 2008, 2009a) and social anxiety (Amir et al., 2003; Mogg et al., 2004; Wieser et al., 2009). These studies generally differ in terms of initial hypervigilance, difficulties in disengagement, or avoidance that they find in the different kinds of fears. This, therefore, raises the question whether one single theoretical account can accommodate research findings on attentional biases in specific phobias and social anxiety.

Our results support the above-mentioned notion in that fear of spiders correlates with the observed initial hypervigilance but this is not the case for social anxiety. This suggests that distinct mechanisms may be underlying the two fears. It will be fruitful for the understanding of both fear domains to disentangle the specific mechanisms in future research. One plausible reason for the differences may in part be due to the intensity of reported fear in our sample. When each one of our groups is compared to normative controls, spider fearful participants report higher levels of spider fear compared to socially anxious participants who score lower on social anxiety (For spider fear: Rinck et al., 2002; for social anxiety: Sosic et al., 2008). One plausible explanation for this is that more socially anxious participants may have avoided participation in the experiment due to their fear. Even though social anxiety is highly prevalent in the population, individuals experiencing this fear are less expected to join experiments compared to spider fearful individuals.

Taken together, the present research is one of the few examples of a direct comparison of different kinds of emotional stimuli. Furthermore, it sheds light on how different categories of threat cues compete for visual attention and how attentional biases are manifested in the context of anxiety. The results suggest that both spiders and angry human faces preferentially guide attentional processes and they seem to be ingrained in our defense system as evolutionary fear-relevant stimuli.

## Author Contributions

EB contributed to the conceptualization and design of the work, data collection, data analysis, writing of the manuscript, and final approval. AG contributed to the conceptualization, programming, design, data analysis, revisions, and final approval. FB contributed to the conceptualization, data analysis, revisions, and final approval. AW contributed to the conceptualization, data analysis, and final approval. GA contributed to the conceptualization, funding, data analysis, continuous revisions, and final approval.

## Conflict of Interest Statement

The authors declare that the research was conducted in the absence of any commercial or financial relationships that could be construed as a potential conflict of interest. The reviewer ADC and handling Editor declared their shared affiliation.

## Footnotes

1 We also included STAI State and Trait scores in the analysis. STAI State did not have any significant effect on the results. While STAI Trait correlates with emotion in the first block. To check whether the effect we find in the first block is due to spider fear or general negative affect we conducted a correlation and regression analysis with FAS scores and STAI Trait scores as potential predictors. The stepwise regression reveals that when the predictor FAS is held constant, the STAI-T is not significant (*p* = 0.16). This suggests that the emotion effect we find in our data is due to the fear of spiders and not the general negative affect.

## References

[B1] Alpers G. W. (2008). Eye-catching: right hemisphere attentional bias for emotional pictures. *Laterality* 13 158–178. 10.1080/13576500701779247 18302058

[B2] Alpers G. W. Adolph D. Pauli P. (2011). Emotional scenes and facial expressions elicit different psychophysiological responses. *Int. J. Psychophysiol.* 80 173–181. 10.1016/j.ijpsycho.2011.01.010 21277913

[B3] Alpers G. W. Gerdes A. B. M. (2007). Here is looking at you: emotional faces predominate in binocular rivalry. *Emotion* 7 495–506. 10.1037/1528-3542.7.3.495 17683206

[B4] Alpers G. W. Gerdes A. B. M. Lagarie B. Tabbert K. Vaitl D. Stark R. (2009). Attention and amygdala activity: an fMRI study with spider pictures in spider phobia. *J. Neural. Transm.* 116 747–757. 10.1007/s00702-008-0106-8 18726545

[B5] Amir N. Elias J. Klumpp H. Przeworski A. (2003). Attentional bias to threat in social phobia: facilitated processing of threat or difficulty disengaging attention from threat? *Behav. Res. Ther.* 41 1325–1335. 10.1016/S0005-7967(03)00039-1 14527531

[B6] Armstrong T. Olatunji B. O. (2012). Eye tracking of attention in the affective disorders: a meta-analytic review and synthesis. *Clin. Psychol. Rev.* 32 704–723. 10.1016/j.cpr.2012.09.004 23059623 PMC3556338

[B7] Berdica E. Gerdes A. B. M. Alpers G. W. (2017). A comprehensive look at phobic fear in inhibition of return: Phobia-related spiders as cues and targets. *J. Behav. Ther. Exp. Psychiatry* 54 158–164. 10.1016/j.jbtep.2016.07.013 27517673

[B8] Berdica E. Gerdes A. B. M. Pittig A. Alpers G. W. (2014). Inhibition of return in fear of spiders: discrepant eye movement and reaction time data. *J. Ophthalmol.* 2014:183924. 10.1155/2014/183924 25101171 PMC4101934

[B9] Blasi G. Hariri A. R. Alce G. Taurisano P. Sambataro F. Das S. (2009). Preferential amygdala reactivity to the negative assessment of neutral faces. *Biol. Psychiatry* 66 847–853. 10.1016/j.biopsych.2009.06.017 19709644 PMC3013358

[B10] Bradley M. Lang P. J. (1994). Measuring emotion: the self-assessment semantic differential manikin and the semantic differential. *J. Behav. Ther. Exp. Psychiatry* 25 49–59. 10.1016/0005-7916(94)90063-9 7962581

[B11] Bublatzky F. Alpers G. W. (2017). Facing two faces: defense activation varies as a function of personal relevance. *Biol. Psychol.* 125 64–69. 10.1016/j.biopsycho.2017.03.001 28267568

[B12] Bublatzky F. Gerdes A. B. M. White A. J. Riemer M. Alpers G. W. (2014). Social and emotional relevance in face processing: happy faces of future interaction partners enhance the late positive potential. *Front. Hum. Neurosci.* 8:493. 10.3389/fnhum.2014.00493 25076881 PMC4100576

[B13] Calvo M. G. Lang P. J. (2004). Gaze patterns when looking at emotional pictures: motivationally biased attention. *Motiv. Emot.* 28 221–243. 10.1023/B:MOEM.0000040153.26156.ed

[B14] Cisler J. M. Koster E. H. W. (2010). Mechanisms of attentional biases towards threat in anxiety disorders: an integrative review. *Clin. Psychol. Rev.* 30 203–216. 10.1016/j.cpr.2009.11.003 20005616 PMC2814889

[B15] Connor K. M. Davidson J. R. T. Erik Churchill L. Sherwood A. Foa E. Weisler R. H. (2000). Psychometric properties of the social phobia inventory (SPIN). New self - rating scale. *Br. J. Psychiatry* 176 379–386. 10.1192/bjp.176.4.379 10827888

[B16] Eisenbarth H. Gerdes A. B. M. Alpers G. W. (2011). Motor-incompatibility of facial reactions: the influence of valence and stimulus content on voluntary facial reactions. *J. Psychophysiol.* 25 124–130. 10.1027/0269-8803/a000048

[B17] Erlich N. Lipp O. V. Slaughter V. (2013). Of hissing snakes and angry voices: Human infants are differentially responsive to evolutionary fear-relevant sounds. *Dev. Sci.* 16 894–904. 10.1111/desc.12091 24118715

[B18] Farah M. J. Wilson K. D. Tanaka J. N. (1998). What is “special” about face perception? *Psychol. Rev.* 105 482–498. 10.1037/0033-295X.105.3.482 9697428

[B19] Fox E. Russo R. Bowles R. Dutton K. (2001). Do threatening stimuli draw or hold visual attention in subclinical anxiety? *J. Exp. Psychol.* 130 681–700. 10.1037//0096-3445.130.4.681 PMC1924776 11757875

[B20] Fox E. Russo R. Dutton K. (2002). Attentional bias for threat: evidence for delayed disengagement from emotional faces. *Cogn. Emot.* 16 355–379. 10.1080/02699930143000527 18273395 PMC2241753

[B21] Gamble A. L. Rapee R. M. (2010). The time-course of attention to emotional faces in social phobia. *J. Behav. Ther. Exp. Psychiatry* 41 39–44. 10.1016/j.jbtep.2009.08.008 19781689

[B22] Gerdes A. B. M. Alpers G. W. Pauli P. (2008). When spiders appear suddenly: spider-phobic patients are distracted by task-irrelevant spiders. *Behav. Res. Ther.* 46 174–187. 10.1016/j.brat.2007.10.010 18154873

[B23] Gerdes A. B. M. Pauli P. Alpers G. W. (2009a). Toward and away from spiders: eye-movements in spider-fearful participants. *J. Neural. Transm.* 116 725–733. 10.1007/s00702-008-0167-8 19156350

[B24] Gerdes A. B. M. Uhl G. Alpers G. W. (2009b). Spiders are special: fear and disgust evoked by pictures of arthropods. *Evolut. Hum. Behav.* 30 66–73. 10.1016/j.evolhumbehav.2008.08.005

[B25] Gerdes A. B. M. Wieser M. J. Muehlberger A. Weyers P. Alpers G. W. Plichta M. (2010). Brain activations to emotional pictures are differentially associated with valence and arousal ratings. *Front. Hum. Neurosci.* 4:175. 10.3389/fnhum.2010.00175 21088708 PMC2982745

[B26] Haxby J. V. Hoffman E. A. Gobbini M. I. (2000). The distributed human neural system for face perception. *Trends Cogn. Sci.* 4 223–233. 10.1016/S1364-6613(00)01482-0 10827445

[B27] Hofmann S. G. Alpers G. W. Pauli P. (2009). “Phenomenology of panic and phobic disorders,” in *Oxford Handbook of Anxiety*, eds Antony M. M. Stein M. B. (Oxford: Oxford University Press), 34–46.

[B28] Holas P. Krejtz I. Cypryanska M. Nezlek J. B. (2014). Orienting and maintenance of attention to threatening facial expressions in anxiety - An eye movement study. *Psychiatry Res.* 220 362–369. 10.1016/j.psychres.2014.06.005 25107319

[B29] Kolassa I. Kolassa S. Musial F. Miltner W. H. R. Schiller F. (2008). Event-related potentials to schematic faces in social phobia. *Cogn. Emot.* 21 1721–1744. 10.1080/02699930701229189

[B30] Lang P. J. Bradley M. M. Cuthbert B. N. (1999). *International Affective Picture System (IAPS): Instruction Manual and Affective Ratings.* Technical Report A-4 Gainesville, FL: University of Florida.

[B31] Langeslag J. E. S. van Strien W. J. (2018). Early visual processing of snakes and angry faces: an ERP study. *Brain Res.* 1687 297–303. 10.1016/j.brainres.2017.10.031 29102778

[B32] Laux L. Glanzmann P. Schaffner P. Spielberger C. D. (1981). *Das State-Trait-Angstinventar (STAI): Theoretische Grundlagen und Handanweisung.* Weinheim: Beltz Test GmbH.

[B33] Lenhard W. Lenhard A. (2014). *Hypothesis Tests for Comparing Correlations.* Bibergau: Psychometrica 10.13140/RG.2.1.2954.1367

[B34] Liang C. Tsai J. Hsu W. (2017). Sustained visual attention for competing emotional stimuli in social anxiety: an eye tracking study. *J. Behav. Ther. Exp. Psychiatry* 54 178–185. 10.1016/j.jbtep.2016.08.009 27569741

[B35] Lipp O. V. (2006). Of snakes and flowers: does preferential detection of pictures of fear-relevant animals in visual search reflect on fear-relevance? *Emotion* 6 296–308. 10.1037/1528-3542.6.2.296 16768561

[B36] Lundqvist D. Flykt A. Öhman A. (1998). *The Karolinska Directed Emotional Faces—KDEF (CD ROM).* Stockholm: Karolinska Institute.

[B37] Macleod C. Mathews A. (1988). Anxiety and the allocation of attention to threat. *Q. J. Exp. Psychol. A* 40 653–670. 10.1080/14640748808402292 3212208

[B38] Mallan K. M. Lipp O. V. Cochrane B. (2013). Slithering snakes, angry men and out-group members: what and whom are we evolved to fear? *Cogn. Emot.* 27 1168–1180. 10.1080/02699931.2013.778195 23556423

[B39] Mathews A. Mackintosh B. (1998). A cognitive model of selective processing in anxiety. *Cogn. Ther. Res.* 22 539–561. 10.1023/A:1018738019346

[B40] Melfsen S. Florin I. (2002). Do socially anxious children show deficits in classifying facial expressions of emotions? *J. Nonverb. Behav.* 26 109–126. 10.1023/a:1015665521371

[B41] Mineka S. Öhman A. (2002). Phobias and preparedness: the selective, automatic, and encapsulated nature of fear. *Biol. Psychiatry* 52 927–937. 10.1016/S0006-3223(02)01669-4 12437934

[B42] Mogg K. Bradley B. P. (1998). A cognitive motivational analysis of anxiety. *Behav. Res. Ther.* 36 809–848. 10.1016/S0005-7967(98)00063-1 9701859

[B43] Mogg K. Bradley B. P. (2006). Time course of attentional bias for fear-relevant pictures in spider-fearful individuals. *Behav. Res. Ther.* 44 1241–1250. 10.1016/j.brat.2006.05.003 16870133

[B44] Mogg K. Philippot P. Bradley B. P. (2004). Selective attention to angry faces in clinical social phobia. *J. Abnorm. Psychol.* 113 160–165. 10.1037/0021-843X.113.1.160 14992669

[B45] Öhman A. (2009). Of snakes and faces: an evolutionary perspective on the psychology of fear. *Scand. J. Psychol.* 50 543–552. 10.1111/j.1467-9450.2009.00784.x 19930253

[B46] Öhman A. Lundqvist D. Esteves F. (2001). The face in the crowd revisited: a threat advantage with schematic stimuli. *J. Pers. Soc. Psychol.* 80 381–396. 10.1037/0022-3514.80.3.381 11300573

[B47] Öhman A. Mineka S. (2001). Fears, phobias, and preparedness: toward an evolved module of fear and fear learning. *Psychol. Rev.* 108 483–522. 10.1037//0033-295X.108.3.483 11488376

[B48] Rinck M. Becker E. S. (2006). Spider fearful individuals attend to threat, then quickly avoid it: evidence from eye movements. *J. Abnorm. Psychol.* 115 231–238. 10.1037/0021-843X.115.2.231 16737388

[B49] Rinck M. Bundschuh S. Engler S. Müller A. Wissmann J. Ellwart T. (2002). Reliabilität und validität dreier instrumentezur messung von angst vor spinnen. *Diagnostica* 48 141–149. 10.1026//0012-1924.48.3.141

[B50] Royston P. Altman D. G. Sauerbrei W. (2006). Dichotomizing continuous predictors in multiple regression: a bad idea. *Statist. Med.* 25 127–141. 10.1002/sim.2331 16217841

[B51] Seligman M. E. P. (1971). Phobias and preparedness. *Behav. Ther.* 2 307–320. 10.1016/S0005-7894(71)80064-3 27816071

[B52] Sosic Z. Gieler U. Stangier U. (2008). Screening for social phobia in medical in- and outpatients with the German version of the Social Phobia Inventory (SPIN). *J. Anxiety Disord.* 22 849–859. 10.1016/j.janxdis.2007.08.011 17923381

[B53] Spielberger C. D. Gorsuch R. L. Lushene R. Vagg P. R. Jacobs G. A. (1983). *Manual for the State-Trait Anxiety Inventory.* Palo Alto, CA: Consulting Psychologists Press.

[B54] Szymanski J. William O. (1995). Fear of spiders questionnaire. *J. Behav. Ther. Exp. Psychiatry* 26 31–34. 10.1016/0005-7916(94)00072-T 7642758

[B55] Weymar M. Gerdes A. B. M. Löw A. Alpers G. W. Hamm A. O. (2013). Specific fear modulates attentional selectivity during visual search: electrophysiological insights from the N2pc. *Psychophysiology* 50 139–148. 10.1111/psyp.12008 23252841

[B56] Wiemer J. Gerdes A. B. M. Pauli P. (2013). The effects of an unexpected spider stimulus on skin conductance responses and eye movements: an inattentional blindness study. *Psychol. Res.* 77 155–166. 10.1007/s00426-011-0407-7 22227916

[B57] Wieser M. J. Pauli P. Weyers P. Alpers G. W. Mühlberger A. (2009). Fear of negative evaluation and the hypervigilance-avoidance hypothesis: an eye-tracking study. *J. Neural Trans.* 116 717–723. 10.1007/s00702-008-0101-0 18690409

